# Mobile health interventions for HIV/STI prevention among youth in low- and middle-income countries (LMICs): a systematic review of studies reporting implementation outcomes

**DOI:** 10.1186/s43058-021-00230-w

**Published:** 2021-11-06

**Authors:** Ucheoma Nwaozuru, Chisom Obiezu-Umeh, Thembekile Shato, Florida Uzoaru, Stacey Mason, Victoria Carter, Sunita Manu, Karan Modi, Jessica Parker, Oliver Ezechi, Juliet Iwelunmor

**Affiliations:** 1 College for Public Health and Social Justice, Saint Louis University, Salus center, 3545 Lafayette Avenue, Saint Louis, Missouri 63104 USA; 2 Brown School, Washington University in Saint Louis, 1 Brookings Drive, Saint Louis, Missouri 63130 USA; 3 Nigerian Institute of Medical Research, 6 Edmund Crescent, Yaba, Lagos Nigeria

**Keywords:** Mobile health, Young people, HIV/STI prevention, Implementation outcomes, Mobile phone, Low- and middle-income countries

## Abstract

**Background:**

Advances and proliferation of technologies such as mobile phones may provide opportunities to improve access to HIV/STI services and reach young people with high risk for HIV and STI. However, the reach, uptake, and sustainability of mobile health (mHealth) HIV/STI interventions targeting young people aged 10–24 years in low- and middle-income countries (LMICs) are largely unknown. To address this gap and to inform implementation science research, a review was conducted to summarize what is known, and what we need to know about implementing mhealth interventions for HIV/STI prevention targeting young people in LMICs.

**Methods:**

We used the Preferred Reporting Items for Systematic Reviews and Meta-Analyses (PRISMA) guidelines for this review. Drawing upon Proctor’s eight implementation outcome measures, we evaluated the acceptability, adoption, appropriateness, cost, feasibility, fidelity, penetration, and sustainability of  m-health HIV/STI interventions targeting young people in LMICs. The search was performed from September 2020–January 2021 and updated on March 1, 2021, in Cumulative Index to Nursing and Allied Health Literature (CINAHL), PubMed, SCOPUS, Global Health, and Web of Science. Eligible studies were required to include an HIV/STI prevention outcome, target young people aged 10–24 years, include a comparison/control group, and reporting of atleast one implementation outcome as outlined by Proctor.

**Results:**

A total of 1386 articles were located, and their titles and abstracts were screened. Of these, 57 full-text articles were reviewed and subsequently, and 11 articles representing 6 unique interventions were included in the systematic review. Acceptability 6 (100%), appropriateness 6 (100%), and feasibility 5(83%) were the most frequently evaluated implementation outcomes. Adoption 2 (33%), fidelity 1 (17%), and cost 1 (17%) were rarely reported; penetration and sustainability were not reported.

**Conclusions:**

This review contributes to implementation science literature by synthesizing key implementation outcomes of mHealth HIV/STI interventions targeting young people in LMICs. Future research is needed on m-health HIV/STI implementation outcomes, particularly the penetration, cost, and long-term sustainability of these interventions. Doing so will enhance the field’s understanding of the mechanisms by which these interventions lead or do not lead to changes in high HIV/STI risk and vulnerability among young people in LMICs.

**Supplementary Information:**

The online version contains supplementary material available at 10.1186/s43058-021-00230-w.

Contributions to the literature

- With recent calls for efforts to enhance the translation of effective interventions into real-world settings, this systematic review examined the implementation outcomes of mhealth interventions for HIV/STI prevention among young people in LMICs. This study addresses an important gap in knowledge given the paucity of evidence regarding the dissemination and scale-up of mhealth interventions for HIV/STI prevention among young people residing in resource-constrained settings.
- This systematic review yielded only six unique comparison-group interventions across eleven articles focused on HIV/STI prevention among young people in LMICs. This suggests that the adoption and delivery of mhealth interventions for HIV/STI prevention among young people in LMICs using rigorous study designs is occurring more gradually than the rapid increase in mobile technologies in the region.
- Findings from this systematic review highlight gaps in the reporting of macro-level implementation outcomes such as penetration and sustainability that can impact the long-term effect of interventions on population health. Future studies can help address this measurement gap by providing nuanced measures for these poorly reported long-term implementation outcomes.

## Background

In the past two decades, there has been an explosive increase in the ownership of mobile phones globally, with over 7 billion people with a mobile phone subscription in 2018 [1]. The global proliferation of mobile phone use is largely driven by rapid uptake in low- and middle-income countries (LMICs) [2–4]. The growing ubiquity and penetration of mobile phone use have led to an increase in the utilization of mobile phones to address public health concerns, broadly referred to as mobile health (mHealth) [5, 6].

MHealth interventions have shown some success in expanding access to care and improving existing health interventions [4, 6–8]. Among young people, the rapid increase in the use of mobile phone technologies affords additional modalities/opportunities to meet their health needs [9, 10]. Human immunodeficiency virus (HIV) and other sexually transmitted infections (STIs) remain significant public health concerns among young people in LMICs [10], suggesting the need for effective strategies to improve access and reach of evidence-based HIV/STI prevention interventions. MHealth interventions may contribute to filling this gap by reaching young people not currently reached by existing HIV/STI interventions.

Previous studies have highlighted ways in which mHealth can promote HIV and STI prevention including serving as reminders for health action, boosters to reinforce sexual risk reduction skills, platforms to promote HIV/STI knowledge, and linkage to HIV/STI screening services [11–13]. Studies evaluating the effectiveness of mhealth interventions have also shown promising results such as increase in HIV testing, condom use, and HIV/STI knowledge among young people [14, 15]. A 2017 systematic review on the use of mobile technologies for adolescent sexual and reproductive health in LMICs concluded that interventions delivered through this modality were effective for enhancing HIV and STI prevention [16]. Mhealth interventions were found to improve HIV/STI knowledge and linkage to STI testing services [16]. Overall, existing evidence suggests that mHealth interventions can promote HIV/STI prevention programs/interventions [15] and that the use of mHealth interventions are generally acceptable for young people [15, 17]. Despite documented impact and popularity of mHealth interventions for HIV and STI prevention, there is limited evidence demonstrating how—and if—such interventions with reported efficacy have been translated beyond research settings and implemented in real-world settings. Given the growing need to enhance the uptake of innovative approaches, such as mHealth interventions for HIV/STI prevention among youth in LMICs, it is critical to identify implementation components that influence translating these research evidence to real-world settings.

Implementation science research may improve the uptake and reach of m-health HIV/STI interventions targeting young people in LMICs. Implementation science research helps to improve the knowledge of factors that influence the effectiveness of an intervention and strategies needed to accelerate the integration of research findings and research-based innovations into real-world practice settings [18]. Proctor and colleagues [18] implementation outcome framework provides a tool for measuring and operationalizing implementation outcomes—acceptability, adoption, appropriateness, cost, feasibility, fidelity, penetration, and sustainability. This tool was designed to help evaluate the components and success of intervention implementation efforts, and it is widely used in implementation science research [18]. Evaluating these implementation outcomes is crucial to gain information on the implementation process and to identify potential barriers and facilitators to intervention translation to real-world settings [18, 19].

While prior systematic reviews have reported that mHealth interventions can impact HIV and STI prevention [2, 20], no published systematic review to our knowledge has examined the implementation outcomes of these interventions. As a result, our study sought to identify and evaluate the implementation outcomes documented in mHealth interventions for HIV and STI prevention among young people in LMICs. These findings may inform the real-world implementation of these interventions particularly among hard-to-reach youth populations in LMICs.

## Methods

The systematic review was conducted and reported according to the Preferred Reporting Items for Systematic Reviews and Meta-Analyses (PRISMA) guidelines [21]. The PRISMA checklist is available in Additional file 1. The protocol is registered on PROSPERO, protocol ID: CRD42020196138.

### Inclusion and exclusion criteria

Articles were included in the review if:

- Researchers used mobile health (mHealth) and addressed HIV/STI prevention in their intervention
- They were full-text peer-reviewed empirical research
- The study design was a randomized controlled trial (RCT) (with comparison group and random assignment) or quasi-experimental (with comparison groups but no random assignment) or stepped-wedge, controlled before-after, or interrupted time series design with a control group
- The intervention was conducted in at least one country classified as LMICs as defined by the World Bank classification of country income groups [22]
- Researchers reported at least one outcome evaluation that assessed the impact of the intervention on at least one or more of the following HIV/STI prevention outcomes among young people: HIV/STI testing, condom use, condom use self-efficacy and attitudes, condom use intentions, HIV/STI related knowledge, PrEP use, number of sexual partners, HIV/STI incidence
- The research targeted adolescents and young people aged 10-24 years. Interventions that were not specific to adolescents and young people but reported separately for this population were eligible for the review.
- Implementation outcome(s) were reported (i.e., acceptability, adoption, appropriateness, cost, feasibility, fidelity, penetration, and sustainability) [18]
- Was published in English language

Articles were excluded in the review if:

- No intervention was performed
- The study did not measure any HIV/STI related prevention outcomes
- mHealth tools were used only for data collection or sample recruitment
- The study did not include a control group. We only included studies with comparison groups in the review to minimize the risk of biases associated with non-comparison group study designs
- The study did not report any implementation outcome
- Were reviews, commentaries, editorials, conference papers, and other non-peer-reviewed publications
- Studies were not available in English

No limitation- or restriction- on the year of publication was applied.

### Search strategy

The literature search was conducted in September 2020 through January 2021 and repeated March 1, 2021, using the following 5 electronic databases: Cumulative Index to Nursing and Allied Health Literature (CINAHL), PubMed, SCOPUS, Global Health, and Web of Science. The searches were performed using the following list of keywords and mesh terms around the four domains of “HIV and STI prevention,” “mobile technology,” “young people,” and “LMICs.” A detailed search strategy for the PubMed database is provided in Additional file 2. Manual reference searches of prior systematic reviews related to mHealth interventions for STI/HIV prevention were completed for other potentially relevant articles [23–27]. We also examined reference lists of all included articles and relevant reviews [20, 24, 28] to search for additional studies.

### Study selection criteria

Following the search, all identified citations were collated and uploaded into Endnote X8 software, where duplicates were removed. Titles and abstracts were screened by two independent reviewers (UN, CO) against the inclusion and exclusion criteria for the review. Studies that met the inclusion criteria were retrieved in full. The full texts of selected studies were retrieved and assessed in detail against the inclusion criteria by two reviewers (UN, CO). Full texts of studies that did not meet the inclusion criteria were excluded and reasons for the exclusion were documented. Disagreements were resolved through discussion between the two reviewers at each stage of the study selection process. The results of the literature search process are presented in a PRISMA flow diagram.

### Data extraction and analysis

Data from the included studies were extracted independently by two reviewers (UN, CO) with a piloted data extraction form. The process was validated by assessing the data extraction form on a small number of randomly selected studies (*n*=3) that were completed independently by the two reviewers. A third person (TS) evaluated the extracted data using the extraction form from the two reviewers (UN, CO) and discussed discrepancies with them.

Data extracted from each study included the year of publication, authors, country of origin, study objective, study design, population and age, participants sample size, intervention description (format, content, setting, mode of delivery), intervention characteristics, outcome measures, and key findings. Implementation outcomes were extracted according to the implementation outcome framework by Proctor and colleagues acceptability, adoption, appropriateness, cost, feasibility, fidelity, penetration, and sustainability [18] and an adapted data extraction tool used by Ugalde and colleagues [29]. Table 1 provides more details on the definition implementation outcomes for data extraction.

**Table 1:** Definition of implementation outcomes for data extraction [18, 29]

Implementation outcome	Definition
Acceptability	Perception among implementation stakeholders that a given treatment, service, practice, or innovation is agreeable, palatable, or satisfactory
Adoption	The intention, initial decision, or action to try or employ an innovation or evidence-based practice
Appropriateness	The perceived fit, relevance, or compatibility of the innovation or evidence-based practice for a given practice setting, provider, or consumer; and/or perceived fit of the innovation to address a particular issue or problem
Feasibility	The extent to which a new treatment, or an innovation, can be successfully used or carried out within a given agency or setting
Fidelity	The degree to which an intervention was implemented as it was prescribed in the original protocol or as it was intended by the program developers
Cost	The financial impact of an implementation effort
Penetration	The integration of a practice within a service setting and its subsystems
Sustainability	The extent to which a newly implemented treatment is maintained or institutionalized within a service setting ongoing, stable operations

Given the heterogeneity of the studies (variety of intervention components and outcomes measures) included in this review, it was not practical to perform a meta-analysis. Therefore, the extracted data were analyzed using narrative synthesis. Details extracted from the included articles were synthesized using tabulation and textual description [30].

First, we performed a descriptive analysis of the final articles to record key study characteristics such as authors, publication year, study aim, study design, sample size, country of origin, types of intervention, and study outcomes. Second, we reported on implementation outcome measures for the included interventions as defined by Proctor and colleagues [18].

### Risk of bias and quality assessment

The Cochrane risk of bias assessment tool was used to assess the risk of selection bias, reporting bias, performance bias, detection bias, and attrition bias in randomized controlled trials (RCTs) [31]. The tool consists of six domains: selection bias, performance bias, detection bias, attrition bias, reporting bias, and other biases [31, 32]. Two reviewers (U.N. and C.O) independently assessed the quality of the selected studies. Any discordance in the assignment of quality assessment was resolved by discussion. The risk of bias of the interventions was rated as low, high, or unclear. The Cochrane Collaboration risk of bias assessment tool was used to evaluate the internal validity of the studies included in the review, and no study was excluded from the review due to the risk of bias assessment score. The relevance of articles included in the review was ascertained by the study selection process according to the pre-defined inclusion and exclusion criteria.

## Results

### Literature search results

**Fig. 1:**
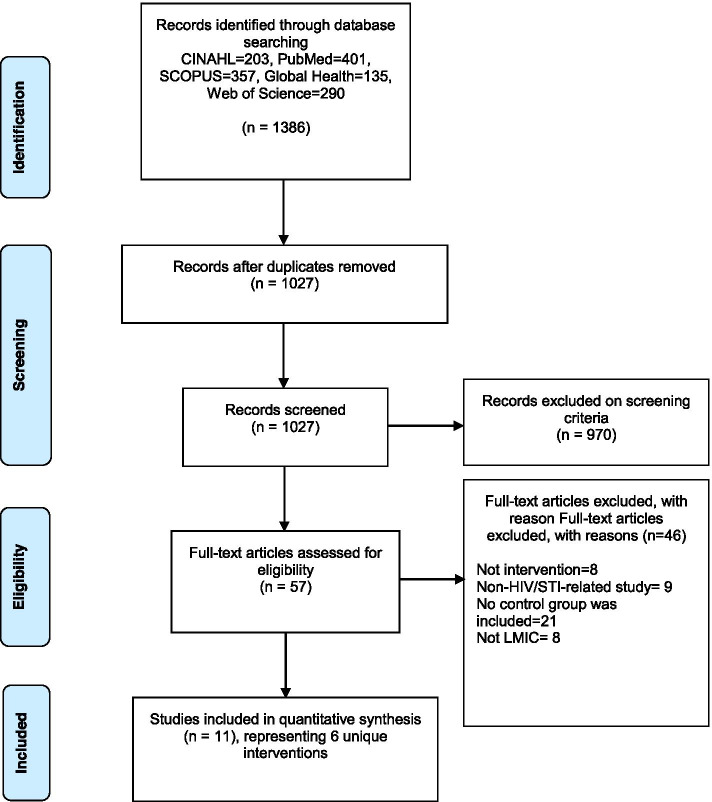
Flow diagram of the search strategy. 11 articles representing 6 interventions were included in the review

Figure 1 presents a flow chart of the literature search and selection process. We identified a total of 1386 studies through the journal database. Of these, 57 articles were potentially relevant based on their title and abstract. The full text of these 57 articles were assessed for eligibility, of which 46 were excluded. The remaining 11 articles were finally included in the review. Reviewing the reference lists of the included papers and previous systematic reviews in the area did not result in additional articles. In total, 11 articles representing 6 unique interventions were included in the review.

### Study characteristics

An overview of the study characteristics of the selected studies is presented in Table 2. Four (67%) of the interventions were conducted in sub-Saharan Africa: 2 (33%) in Kenya [33, 34], 1 (17%) in Ghana [15], and 1 (17%) in Uganda [35]. Two (33%) interventions were in East Asia: both of them in China [36, 37]. The average number of participants included in the studies was 594, ranging from 60 [33] to 1337 [36].

**Table 2:** Characteristics of included 6 interventions across 11 articles included in the review

Author(s), Year	Country/study setting/study design	Participants (sample size, gender, age)	Implementation/study objective	Intervention description (features, duration)	Primary outcomes	Main results
Lou et al. 2006 [36]	Country: China Setting: School-based (two high schools and four colleges) Study design: Quasi-experimental (non-randomized intervention and control groups)	Sample size: 1337 participants (624 in the intervention group, and 713 in the control group) Gender: Males and Females Age: 14–24 years	To examine intervention effect on adolescent and young people HIV/STI-related knowledge and changes inattitudes and behaviors	Intervention group: participants received access to a web-based intervention that offered sexual and reproductive health knowledge, service information, counseling, and discussion. Control group: No special sexual education was provided. Received their usual care mHealth Component: Web application Duration: 10 months	HIV and STI knowledge, attitude score, and proportion of sex-related behaviors	The median scores of the overall knowledge on each specific aspect of reproductive health such as reproduction, contraception, condom, sexually transmitted infections (STIs), and human immunodeficiency virus/acquired immune deficiency syndrome (HIV/AIDS) were significantly higher in the intervention group compared with those in the control group at post-intervention (*p* < 0.0001)
Odeny et al., 2012 [38]; Odeny et al. 2014 [34]	Country: Kenya Setting: Clinic-based Study design: Two-arm parallel-group RCT	Sample size: 1200 participants (600 in the intervention group, and 600 in the control group) Gender: Males only Age: 18–20 years	To examine the effect of text messaging to deter resumption of sex before 42 days post-circumcision, and post-operative clinic visit after circumcision	Intervention group: participants received usual care (which consisted of HIV testing and counseling, screening and treatment for STIs, condom promotion and provision, risk reduction, and safe sex counseling, the male circumcision procedure and post-operative review) and SMS about post-operative care, appointment reminders and healthy sex behaviors (including abstinence) for the first 7 days and on days 8, 14, 21, 28, 35, 41, and 42 post-procedure Control group: participants received usual care (which consisted of HIV testing and counseling, screening and treatment for sexually transmitted infections, condom promotion and provision, risk reduction, and safe sex counseling, the MC procedure, and postoperative review) only mHealth Component: SMS Duration: 2 months	Health-seeking behavior of clinic attendance	Increase in sexual and reproductive health clinic visits among participants in the intervention groupcompared to those in the control group
Rokicki et al. 2017 [15]; Rokicki & Fink, 2017 [14]	Country: Ghana Setting: School-based Study design: RCT	Sample size: 498 participants (10 schools in the intervention group consisting of 205 participants, and 12 schools in the control group consisting of 291 participants) Gender:Females only Age: 14-24 years	To assess the reach of the intervention among the target population and intervention effect on sexual and reproductive health knowledge	Intervention group: Participants received an interactive mobile phone quiz game where participants could win mobile phone credit by texting correct answers to SRH questions. The messages focused on pregnancy prevention and contained information on topics of reproductive anatomy, pregnancy, STIs, and contraception including male and female condoms, birth control pills, and emergency contraception. Control group: Received one message each week with information about malaria mHealth Component: SMS Duration: 12 weeks	Increase in sexual and reproductive health knowledge	81% of participants engaged with the mHealth intervention. The intervention was effective at increasing knowledge of sexual reproductive health across all strata. Higher levels of engagement were associated with higher knowledge scores up to a year later. Participants in the intervention group who were sexually active reported lower odds of self-reported pregnancy from baseline to 15 months
Winskell et al. 2018 [33]; Sabben et al. 2019 [44]	Country: Kenya Setting: Community-based (participants were recruited from schools) Study design: RCT	Sample size: 60 participants (30 participants in the intervention group and 30 participants in the control group) Gender: Males and Females Age: 11–14 years	The determine the influence of the intervention on increased age of sexual debut and condom use at sexual debut	Intervention group: participants received*Tumaini,* a narrative-based game for android smartphones. The game comprisesapproximately 12 h of discrete gameplay and is designed to be replayed so that players can observe the outcomes of the different decisions. The game was designed to increase age and condom use at first sex by increase knowledge about sexual health and HIV; building risk-avoidance and risk-reduction skills and related self-efficacy; challenging HIV stigma and harmful gender norms and attitudes; fostering future orientation, goal setting, and planning, and promoting dialog with adult mentors Control group: Received standard of care, no additional intervention beyond any existing sex education from family, school, and peers mHealth Component: Mobile application Duration: 16 days over 3-week school holiday period	Increase condom use at sexual debut, increase sexual health-related knowledge	Participants in the intervention arm showed significant gains in sexual health-related knowledge and self-efficacy compared to participants in the control arm at 6-week postintervention completion
Ybarra et al. 2012 [39]; Ybarra et al. 2013 [40]; Ybarra et al. 2014 [35]	Country: Uganda Setting: School-based Study design: RCT	Sample size: 366 participants (183 participants in the intervention group and 183 participants in the control group) Gender: Males and females Age: 12 years and older	To increase abstinence and/or condom use	Intervention group: Participants received CyberSenga five 1-h online modules (+ booster module), tailored for gender and culture. The topics covered included (1) information about HIV (e.g., what is HIV and how is it prevented), (2) decision making and communication (e.g., steps to solving a problem; strategies for communicating your solution to others assertively), (3) motivations to be healthy (e.g., reasons why adolescents choose to be abstinent versus to have sex), (4) how to use a condom to be healthy (e.g., demonstration of correct condom use, testimonials from people similar to the participants who used condoms), (5) healthy relationships (e.g., components of healthy relationships; strategies to address coercive gifts), and (6) review. Control group: Participants received “treatment as usual” (i.e., school-delivered sexuality programming) mHealth Component: Web application (Website) Duration: 12 weeks	Increase condom use, promote abstinence at 3-month post-intervention	At 3-month post-intervention: Abstinence–intervention versus comparison group: 81% vs. 74%, *p* = 0.08); unprotected sex—no difference. At 6-month post-intervention: no significant differences in the main outcomes. Abstinence, I booster group vs. I no booster vs. C: (80% vs. 57% vs. 55%).
Zhu et al. 2019 [37]	Country: China Setting: Community (recruited through WeChat) Study design: RCT	Sample size: 100 participants (50 participants in the intervention group and 50 participants in the control group) Gender: Males only Age: 18–29 years	To examine preliminary effects of interventions on HIV testing, and use of the HIVST kits, and condom use	Intervention group: participants received two oral HIVST kits and access to *WeTest*, a WeChat group that provided app-based messages and referrals to health services related to HIV. Control group: Participants received two oral HIVST kits only mHealth Component: Mobile application Duration: 4 weeks	HIV testing, condom use	Participants in the intervention group had significantly higher rates of HIV testing (adjusted rate ratio RR=1.99, 95% confidence interval (CI) 1.07–3.84) and higher rates of testing via oral HIVST (adjusted RR=2.17, 95% CI 1.08–4.37) compared to participants in the control group.

The mHealth components across the six interventions were delivered using three modalities: (1) as mobile applications, (2) as phone-based short message services (SMS), and (3) as web-based application. Specifically, two interventions used mobile phone applications to provide HIV/STI prevention services and information [33, 37]. One of the intervention was delivered as a narrative-based game for android smartphones [33] and one used *WeChat—*a messaging mobile application [37]. *WeChat* was used to provide messages and referrals to health services. Two other interventions used web-based applications to deliver intervention components [35, 36]. Lou et al. [36] utilized a web-based application to offer sexual and reproductive health knowledge, service information, counseling, and discussion to study participants and Ybarra and colleagues [35] utilized a web-based application called *CyberSenga* to provide online module information on HIV prevention and risk-reduction education. Additionally, two of the interventions used SMS technology to deliver intervention components [15, 34].

Five of the six (83%) interventions in the review were evaluated through the use of RCT and [15, 33–35, 37], one (17%) using a quasi-experiment [36]. The comparison conditions for five of the interventions were standard of care, access to usual HIV/STI education at school, community, or home [15, 33–36]. For the study focused on promoting HIV self-testing (HIVST) in China, the control condition was the absence of the *Wechat* mobile application which was available to participants in the intervention group [37]. Participants in both the control group and the intervention group received oral HIVST kits for this intervention [37].

### Evidence on the effect of mHealth intervention on HIV/STI prevention

Three of the interventions primarily targeted increasing condom use [33, 35, 37], two targeted improving sexual and reproductive health knowledge [15, 36], and one study targeted delaying sexual intercourse after male circumcision [34]. The various interventions in general focused on different measures of HIV/STI prevention. This included increase in HIV/STI knowledge, reduction in risky sexual behaviors (e.g., delay in sexual intercourse after male circumcision, increase condom use, delay sexual debut), and increase HIV self-testing.

### Implementation outcomes

**Fig. 2:**
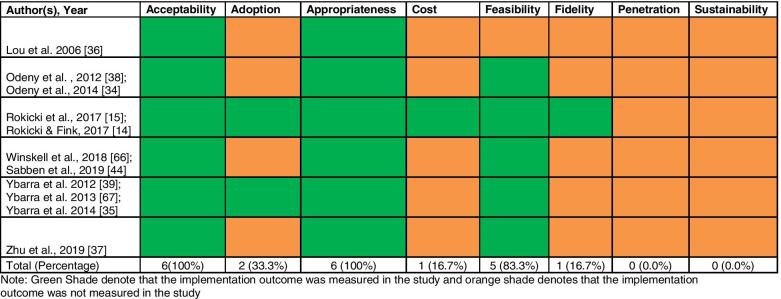
Implementation outcomes reported in 6 interventions across 11 articles included in the review. Note: Green Shade denotes that the implementation outcome was measured in the study and orange shade denotes that implementation outcome was not measured in the study

We identified studies that reported implementation outcomes as defined by Proctor et al. [18]. Figure 2 displays the frequency of which these implementation outcomes were measured across the six interventions included in this review. The most commonly evaluated implementation outcomes across the interventions were acceptability (100%) and appropriateness (100%), followed by feasibility (83.3%). Across all interventions, adoption (33.3%), fidelity (16.7%), and cost (16.7%) were the least measured implementation outcomes. Penetration and sustainability were not evaluated in any of the interventions included in this review. Reporting of implementation outcomes is at the intervention-level for the results provided.

### Acceptability

Acceptability was measured and reported in all six (100%) interventions included in the review [15, 33–37]. Acceptability was identified as an implementation outcome in each study if the study participants indicated satisfaction with the technology or based on their experience with the intervention process. All the interventions were rated to be highly acceptable by study participants, stakeholders, and from reports by the researchers.

Acceptability was evaluated based on data collected on intervention satisfaction from participants’ perspectives for the six interventions. Two of the interventions explicitly measured intervention acceptability using Likert scale survey questionnaires and qualitative feedbacks from study participants [15, 33]. Odeny and colleagues measured acceptability based on the success of SMS used to deliver intervention components to study participants [34]. Intervention acceptability in the studies by Odeny and colleagues in Kenya [34, 38], and Lou and colleagues in China [36] were determined through pilot studies among the target population prior to the intervention implementation. In the intervention by Rokicki et al. [15], acceptability was assessed based on high levels of participants’ engagement in the study. In the intervention in Kenya by Winskell and colleagues [33], study participants found the games to be valuable. Specifically, all participants indicated that they learned a lot from the game and that the information acquired would be very useful in their future. Additionally, 97% of the participants indicated that they would tell their friends to play the game.

All the intervention reported either involved participants and/or key stakeholders in the intervention development process. Lou et al. [36] included participants’ suggestions in the website development process to ensure that it was attractive to the target population (young students). In the intervention by Ybarra et al. [35], young people were involved in the intervention development. They provided feedback on the mobile application usability prior to deployment. The intervention by Zhu et al. [37] engaged participants through interviews to explore users’ preferences concerning content for HIV self-testing promotion. In addition, in the development of Tumaini narrative-based game application adolescents and their parents were convened to provide feedback on the game components to ensure they were suitable for and acceptable to young people in Kenya [33]. To enhance the acceptability of the SMS quiz intervention for adolescents in Ghana, adolescents were engaged in focus group discussions to understand their priority sexual and reproductive health needs. This informed the message content for the intervention, which was further approved by the Health Promotion Unit at the Ghana Health Service [14, 15]. Overall participants and stakeholders’ feedback and suggestions were incorporated in the intervention components and implementation to enhance intervention acceptability.

### Adoption

Adoption defined here as the intention, initial decision, and action to take up or utilize the mHealth intervention was reported in two (33%) interventions [15, 35]. This was only measured at the participants’ level and not at the setting level. Rokicki & Fink [15] evaluated intervention adoption based on high participants’ engagement. Specifically, they measured the total number of times participants replied to the weekly message quiz questions posed in the interactive mobile phone quiz game. Ybarra et al. [35] measured adoption by the number of participants who completed the intervention. The intervention was completed by 95% of participants in the intervention group. For the two interventions that reported on adoption, this was assessed to be high.

### Appropriateness

Appropriateness defined here as the perceived fit, relevance, usefulness, and compatibility of mHealth technology with study participants’ settings and needs was measured in all six (100%) interventions [15, 33–37].Three of the interventions employed strategies to target specific youth populations who may have higher risks for HIV/STI infections compared to their peers. For instance, the intervention by Zhu and colleagues [37] utilized purposive sampling to target men who have sex men (MSM) who were at increased risk for HIV. Similarly, the interventions by Lou et al. [36] and Winskell et al. 2018 [33] recruited study participants using purposive sampling to maximize reach to their target audience. Appropriateness was mainly evaluated based on participants’ feedback. In the intervention by Winskell et al. [33], study participants reported in surveys that the intervention was useful and applicable to preventing HIV and other STIs. They futher indicated that they acquired useful information from the study which were relevant to their daily lives and future. Similarly, participants found the CyberSenga to be acceptable and not contradictory to local norms in Uganda in most cases, however concerns on the the program including a lot of discussion on sex and condoms were raised [35]. Particularly, about 70% of the participants stated that the CyberSenga program "talked too much about sex and condom use". However, evaluation of the program found that information about condom use and sex education was not confusing and contradictory to youth participants who were abstinent [35]. They information was helpful to build risk-reduction skills. The CyberSenga intervention by Ybarra and colleagues [39, 40] also measured intervention appropriateness and fit based on the availability of internet computer access or electricity in the classrooms–the intervention sites. While the intervention components were evaluated to be appropriate in two intervention sites, the fit was limited for sites that did not have Internet or computer access, or electricity. The researchers created mobile cafés for participants in these schools to have access to the intervention components. Overall, their was mixed-reporting of appropriateness across the interventions.

### Feasibility

Feasibility was measured in five (83%) of the interventions [15, 33–35, 37] and defined here as the extent to which the intervention was successfully implemented. This implementation outcome was evaluated based on feedback from study participants and process evaluation of the intervention implementation. For instance, the intervention by Ybarra et al. [35] assessed intervention feasibility using field notes and process measures of the intervention implementation. The intervention was reported to be feasible due to high participation rate. The participation rate for the intervention remained high despite some interruptions in interventions due to conflicting school and participants’ schedules. Given that the intervention *CyberSenga* program required Internet, the absence of Internet interruptions during intervention delivery was reported as an indicator of high intervention feasibility. Zhu et al. [37] measured intervention feasibility based on increased uptake of HIV testing among study participants. In addition, the five interventions all reported on participants eligibility criteria, tscreening process, and the number of participants who finally enrolled in the interventions. These are also important indicators to assess intervention feasibility. Overall, the five interventions were evaluated to be suitable and practical for everyday use for participants.

### Fidelity

Only one (17%) intervention explicitly evaluated intervention fidelity [14]. Fidelity was measured by recording the delivery of sexual reproductive health text messages by staff, to ensure consistency in the messages for intervention participants.

### Penetration

Penetration which is defined as the spread within an eligible population or level of institutionalization [18] was not evaluated in any of the interventions (0%).

### Cost

Cost was reported in one of the interventions [15], defined as the financial impact needed for the delivery of intervention components [18]. The text-messaging intervention for adolescent girls in Ghana reported that the marginal cost for the interactive program per participant was US$1.91 and for the unidirectional program was US$0.30 [15]. The unidirectional intervention sent participants text messages with reproductive health information. The interactive intervention engaged adolescents in text-messaging reproductive health quizzes [15]. The authors explained this marginal cost to be inexpensive and have the potential to reach a large and diverse population [15]. However, additional information was not provided on how the cost was computed or what the cost consisted of.

### Sustainability

None of the intervention evaluated sustainability, which is defined as the extent to which an intervention can be maintained, routinized, or institutionalized by a provider or facility [18].

#### Quality of evidence

The quality assessment of the selected articles is reported in Table 3. Overall, the methodological rigor of the included interventions was high. The level of bias did not vary widely, the majority of the interventions (86%) had a bias rate of 14.3%, and only one of the interventions had a bias rate of 28.6%. The major strengths of the interventions included the use a random selection process and random assignment of participants to the intervention components, detailed description of intervention and participants’ characteristics. The low attrition reported across the interventions was another major strength. The major limitation of the interventions was that study participants were not blinded to the intervention allocation. According to the intervention by Rokicki and colleagues [15], it was not feasible and practical to blind the study participants to the intervention given the overt participation nature of the mhealth intervention. In the intervention by Winskell and colleagues [33], the researcher was blinded to the allocation while study participants were revealed to their assignment as randomization.

**Table 3:** Reporting on quality of included interventions (6 interventions reported in 11 articles included in the review

Author(s), Year	Selection bias (random sequence generation)	Selection bias (allocation concealment)	Performance bias	Detection bias (incomplete outcome data)	Reporting bias (selective reporting)	Other sources of bias	% risk
Lou et al. 2006 [36]	Unclear	High risk	Low risk	Low risk	Low risk	Low risk	28.6%
Odeny et al. 2012 [38]; Odeny et al. 2014 [34]	Low risk	High risk	Low risk	Low risk	Low risk	Low risk	14.3%
Rokicki et al. 2017 [15]; Rokicki & Fink, 2017 [14]	Low risk	High risk	Low risk	Low risk	Low risk	Low risk	14.3%
Winskell et al. 2018 [33]; Sabben et al. 2019 [44]	Low risk	High risk	Low risk	Low risk	Low risk	Low risk	14.3%
Ybarra et al. 2012 [39]; Ybarra et al. 2013 [40]; Ybarra et al. 2014 [35]	Low risk	High risk	Low risk	Low risk	Low risk	Low risk	14.3%
Zhu et al. 2019 [37]	Low risk	High risk	Low risk	Low risk	Low risk	Low risk	14.3%

## Discussion

The current study examined the extent to which mHealth interventions for HIV/STI prevention among young people in LMICs reported on implementation outcomes: acceptability, adoption, appropriateness, cost, feasibility, fidelity, penetration, and sustainability. Findings from this review show variations in the evaluation of these implementation outcomes across the six interventions included in the review. At least one implementation outcome was reported in all six interventions, with each intervention reporting between two to five implementation outcomes. These results suggest that although LMICs are experiencing exponential technological growth with young people increasingly having access to mobile phones, implementation science research in the area of mHealth interventions for HIV/STI prevention in the region may be at a nascent phase and require additional work.

We found several similarities when comparing our results to previous systematic reviews on implementation outcomes. Similar to previous reviews on implementation outcomes in LMICs, acceptability, appropriateness, and feasibility were the most frequently reported implementation outcomes [41–43]. All six interventions in the review reported high intervention acceptability. This was largely evaluated based on self-reports and discussions with study participants and target populations. In addition, stakeholders beyond the study participants such as parents and community stakeholders were also involved to evaluate intervention acceptability. For instance, the interventions by Winskell et al. [33] and Rokicki et al. [15] involved other key stakeholders who provided valuable feedback to optimize for the acceptability and suitability of the intervention components. For the *Tumaini* smartphone application [33], adolescents and their parents were engaged to measure intervention acceptability and in the Rokicki study [15] government stakeholders were also involved to review and approve the intervention message contents.

Further, there were also mixed evidence on the appropriateness of the interventions. While most of the studies reported that mhealth HIV/STI prevention interventions were relevant to and suitable for young people, participants in the *Tumaini intervention* in Kenya found some components of the intervention to be uncomfortable and not age appropriate [44]. Similar concerns on the discussions on sex and condom use were raised for the *CyberSenga* program in Uganda [35]. This suggests the need to ensure that interventions are tailored to be contextually and developmentally appropriate among target populations while developing and implementing intervention contents geared towards HIV/STI prevention among young people. The literature on the adaptation of HIV/STI prevention interventions have highlighted the importance of considering local contexts and cultures to ensure the interventions are culturally and developmentally appropriate [45–47]. Since culture and social norms play a significant role in the acceptance of intervention content around sensitive topics related to sexual practices and sexual risk-reduction practices such as condom use [48–50], these considerations are critical to enhancing intervention adoption and acceptance [51, 52].

In addition, adoption and fidelity were minimally reported in the included interventions. Adoption was measured using various metrics across the interventions. The intervention by Rokicki & Fink [15] measured adoption based on participants’ engagement in the intervention through weekly responses to the mobile-based SMS preventive HIV and STI quizzes while Ybarra et al. [35] evaluated adoption as intervention components completion rate. This finding is consistent with previous studies, suggesting variations in measuring, reporting, and analyzing adoption [18, 53]. However, none of the studies examined setting-level adoption metrics such as location or practice setting readiness or intent-to-use for mHealth interventions to promote HIV/STI prevention. While the information on participant-level adoption are useful, it does not provide nuanced and robust understanding of setting-level intent to try or use m-Health interventions. Future studies should examine setting-level adoption measures, to delineate factors associated with the uptake of the interventions by health facilities, staff, or communities in LMICs. Setting-level information in addition to end-user input provides robust details necessary for designing and adapting interventions to be context-specific [54]. In addition, there was low reporting on intervention fidelity or descriptions on how the interventions are implemented which are crucial information for ensuring the successful replication or adoption of these interventions to maximize effectiveness and public health gains [55]. These gaps are in congruence with reviews of interventions in LMICs, which calls for more robust and consistent measurement of adoption [41, 42, 56] and fidelity [42]. Future reporting of mHealth interventions for young people in LMICs should consider providing detailed fidelity measures or information to provide structure for effective planning, implementation, and evaluation of interventions. As implementation research advances in the area of mhealth applications in LMICs, it is critical to develop a methodological framework that provides a rigorous measurement of fidelity dimensions such as consistency, satisfaction, and quality can be explored [57]. Such implementation science methodologies may provide more nuanced and reliable measures of fidelity that can be adapted for each setting.

Findings from this review also highlight a persistent gap in the measurement of penetration and sustainability, as none of the studies included in the review reported on these implementation outcomes. These implementation outcomes are important broader contextual determinants of policies and strategies for the integration of mHealth interventions in health settings [58–60]. The lack of reporting on penetration and sustainability may be as a result of most studies focusing on preliminary efficacy/effectiveness of these m-health interventions. Nonetheless, to optimize the translation of interventions to real-world settings, it is imperative to assess these implementation outcomes. Penetration and sustainability measure and illuminate practice-level and contextual factors that enhance opportunities for integration of interventions into practice and scale-up [61, 62]. Also, only one of the interventions reported on intervention cost, evidence on the cost, and cost-effectiveness of mobile health interventions are essential to justify scale-up and allocations of funds in regions where resources are scarce [63]. Reporting on cost should be rigorous to account for the financial impact of the mhealth implementation from the cost of mhealth platform to the cost of staff. This should also highlight startup and sustainability costs.

This review must also be seen in the light of some limitations. First, it is possible our search strategy may have excluded potentially eligible articles. To minimize this risk, articles were retrieved from multiple electronic databases and supplemented by manual search and reference list checking of included articles. Second, we limited our review to only RCTs and quasi-experimental studies. Observation studies and non-control studies may provide important information useful for understanding the implementation outcomes of mHealth interventions for HIV/STI prevention among young people in LMICs [64]. Our inclusion criteria may have also resulted in omitting pre-implementation acceptability or feasibility assessments that were not accompanied by RCTs or quasi-experimental studies. However, we opted for interventions that had some comparison groups to minimize the risk of bias associated with non-comparison group study designs. Third, this review assessed the implementation potential of intervention based on the reporting on the published intervention, but this may not mean that it has not been translated into practice. Studies may show limited implementation potential according to reported information in the articles but may have been successfully implemented into practice [29]. Nonetheless, the data extraction and validation by the two reviewers showed no discrepancy in the allocation of implementation outcomes for each study.

Despite the aforementioned limitations, there are several strengths to this systematic review. To our knowledge, this is the first review to elucidate implementation outcomes of mHealth interventions for HIV/STI preventions among young people in LMICs. Findings from this review can inform mHealth implementations in the region, especially for sustainable and large-scale implementations. In addition, key stakeholders and researchers seeking to understand implementation outcomes of mHealth intervention can benefit from the findings of this review [65], as future research questions on how to improve measurements of implementation outcomes and decision-making on effective means to translate evidence from mHealth interventions to practice can be identified.

This systematic review also has implications for implementation science and practice. Findings underscore the importance of documenting implementation outcomes in more detail to inform other researchers interested in implementing HIV/STI prevention mHealth interventions for young people in LIMICs. This review highlights that gaps exist in this area of research. Assessment of implementation outcomes provides a solid framework to assess the implementation of interventions and offer a unique contribution to the field of implementation science. Findings from this study also provide insights into strategies for integrating mHealth interventions for HIV/STI prevention for young people in real-world settings. Through highlighting measured implementation outcomes, we aim to provide evidence to assist researchers, practitioners, and policymakers in the process of planning, reporting, and selecting m-health HIV/STI interventions targeting young people on a larger scale.

## Conclusion

Implementation science has the potential to support the delivery and dissemination of HIV/STI prevention mHealth interventions in LMICs. Notably, this review demonstrates the acceptability, appropriateness, and feasibility of mHealth interventions to promote HIV/STI prevention among young people in LMICs. However, more research is needed in this area to evaluate setting-level adoption, fidelity, widespread penetration, cost, and sustainability. Doing so will enhance the field’s understanding of the mechanisms by which these interventions lead or do not lead to changes in high HIV/STI risk and vulnerability among young people in LMICs.

## Supplementary Information

**Additional file 1.**

**Additional file 2.**

## Data Availability

Not applicable

## Abbreviations

FU: Florida Uzoaru
HIV: Human immunodeficiency virus
JI: Juliet Iwelunmor
JP: Jessica Parker
KM: Karan Modi
LMIC: Low- and middle-income countries
MeSH: Medical subject headings
mHealth: Mobile Health
MSM: Men who have sex with men
OE: Oliver Ezechi
PRISMA: Preferred Reporting Items for Systematic Reviews and Meta-Analyses
RCT: Randomized control trial
SM: Stacey Mason
SMa: Sunita Manu
STI: Sexually transmitted infections
TS: Thembekile Shato
UN: Ucheoma Nwaozuru
VC: Victoria Carter

## References

[1] Mobile cellular subscriptions [https://data.worldbank.org/indicator/IT.CEL.SETS]

[2] Forrest JI, Wiens M, Kanters S, Nsanzimana S, Lester RT, Mills EJ (2015). Mobile health applications for HIV prevention and care in Africa. Curr Opinion HIV AIDS.

[3] ITU releases 2018 global and regional ICT estimates [https://www.itu.int/en/mediacentre/Pages/2018-PR40.aspx]

[4] Mechael PN (2009). The case for mHealth in developing countries. Innovations Technol Governance Globalization.

[5] Sloninsky D, Mechael P (2008). Towards the development of an mhealth strategy: a literary review.

[6] Consulting VW (2009). mHealth for development: the opportunity of mobile technology for healthcare in the developing world.

[7] Kahn JG, Yang JS (2010). Kahn JS: ‘Mobile’health needs and opportunities in developing countries. Health Affairs.

[8] Akter S, Ray P (2010). mHealth-an ultimate platform to serve the unserved. Yearbook Med Informatics.

[9] Higgs ES, Goldberg AB, Labrique AB, Cook SH, Schmid C, Cole CF, Obregón RA (2014). Understanding the role of mHealth and other media interventions for behavior change to enhance child survival and development in low-and middle-income countries: an evidence review. J Health Commun.

[10] Kalamar AM, Bayer AM, Hindin MJ (2016). Interventions to prevent sexually transmitted infections, including HIV, among young people in low-and middle-income countries: a systematic review of the published and gray literature. J Adolescent Health.

[11] Young SD, Cumberland WG, Nianogo R, Menacho LA, Galea JT, Coates T (2015). The HOPE social media intervention for global HIV prevention in Peru: a cluster randomised controlled trial. Lancet HIV.

[12] Zhao Y, Zhu X, Pérez AE, Zhang W, Shi A, Zhang Z, Gao P, Wang J, Yang C, Zaller N (2018). MHealth approach to promote Oral HIV self-testing among men who have sex with men in China: a qualitative description. BMC Public Health.

[13] Cole-Lewis H, Kershaw T (2010). Text messaging as a tool for behavior change in disease prevention and management. Epidemiol Reviews.

[14] Rokicki S, Fink G (2017). Assessing the reach and effectiveness of mHealth: evidence from a reproductive health program for adolescent girls in Ghana. BMC Public Health.

[15] Rokicki S, Cohen J, Salomon JA, Fink G (2017). Impact of a text-messaging program on adolescent reproductive health: a cluster–randomized trial in Ghana. Am J Public Health.

[16] Ippoliti NB, L’Engle K (2017). Meet us on the phone: mobile phone programs for adolescent sexual and reproductive health in low-to-middle income countries. Reproductive Health.

[17] Merrill J, Hershow R, Gannett K, Barkley C: Pretesting an mHealth intervention for at-risk adolescent girls in Soweto, South Africa: studying the additive effects of SMSs on improving sexual reproductive health & rights outcomes. In: Proceedings of the Sixth International Conference on Information and Communications Technologies and Development: Notes-Volume 2: 2013: ACM; 2013: 96-99.

[18] Proctor E, Silmere H, Raghavan R, Hovmand P, Aarons G, Bunger A, Griffey R, Hensley M (2011). Outcomes for implementation research: conceptual distinctions, measurement challenges, and research agenda. Administration and Policy in Mental Health and Mental Health Services Research.

[19] Görlach MG, Schrage T, Bokemeyer C, Kröger N, Müller V, Petersen C, Betz CS, Krüll A, Schulz H, Bleich C (2020). Implementation analysis of patient reported outcomes (PROs) in oncological routine care: an observational study protocol. Health Quality Life Outcomes.

[20] Catalani C, Philbrick W, Fraser H, Mechael P, Israelski DM (2013). mHealth for HIV treatment & prevention: a systematic review of the literature. The open AIDS J.

[21] Moher D, Liberati A, Tetzlaff J, Altman D (2009). Group TP, Oxman A, Cook D, Guyatt G, Swingler G, Volmink J, Ioannidis J, Young C, Horton R, et al. Preferred Reporting Items for Systematic Reviews and Meta-Analyses.

[22] Bank W: World Bank country and lending groups. In.: World Bank Data Help Desk Washington (DC); 2017.

[23] Widman L, Nesi J, Kamke K, Choukas-Bradley S, Stewart J (2018). Technology-based interventions to reduce sexually transmitted infections and unintended pregnancy among youth. J Adolescent Health.

[24] L’Engle KL, Mangone ER, Parcesepe AM, Agarwal S, Ippoliti NB (2016). Mobile phone interventions for adolescent sexual and reproductive health: a systematic review. Pediatrics.

[25] Ihesie CA (2015). Is mobile health (mHealth) the magic bullet? A short review of the impact of mHealth on adolescent sexual health. J Public Health Epidemiol.

[26] Chigona W, Nyemba M, Metfula A: A review on mHealth research in developing countries. J Community Informatics 2012, 9(2).

[27] Muessig KE, Nekkanti M, Bauermeister J, Bull S, Hightow-Weidman LB (2015). A systematic review of recent smartphone, Internet and Web 2.0 interventions to address the HIV continuum of care. Current HIV/AIDS Reports.

[28] Burns K, Keating P, Free C (2016). A systematic review of randomised control trials of sexual health interventions delivered by mobile technologies. BMC Public Health.

[29] Ugalde A, Gaskin CJ, Rankin NM, Schofield P, Boltong A, Aranda S, Chambers S, Krishnasamy M, Livingston PM (2019). A systematic review of cancer caregiver interventions: appraising the potential for implementation of evidence into practice. Psycho-Oncology.

[30] Popay J, Roberts H, Sowden A, Petticrew M, Arai L, Rodgers M, Britten N, Roen K, Duffy S (2006). Guidance on the conduct of narrative synthesis in systematic reviews. Product ESRC Methods Programme Version.

[31] Higgins JP, Green S: Cochrane handbook for systematic reviews of interventions, vol. 4: Wiley; 2011.

[32] Higgins JP, Altman DG, Gøtzsche PC, Jüni P, Moher D, Oxman AD, Savović J, Schulz KF, Weeks L, Sterne JA (2011). The Cochrane Collaboration’s tool for assessing risk of bias in randomised trials. Bmj.

[33] Winskell K, Sabben G, Akelo V, Ondeng'e K, Obong'o C, Stephenson R, Warhol D, Mudhune V (2018). A smartphone game-based intervention (Tumaini) to prevent HIV among young Africans: pilot randomized controlled trial. JMIR mHealth and uHealth.

[34] Odeny TA, Bailey RC, Bukusi EA, Simoni JM, Tapia KA, Yuhas K, Holmes KK, McClelland RS: Effect of text messaging to deter early resumption of sexual activity after male circumcision for HIV prevention: a randomized controlled trial. J Acquired Immune Deficiency Syndromes (1999) 2014, 65(2):e50. 10.1097/QAI.0b013e3182a0a050 PMC3867588 23846561

[35] Ybarra ML, Bull SS, Prescott TL, Birungi R (2014). Acceptability and feasibility of CyberSenga: an Internet-based HIV-prevention program for adolescents in Mbarara, Uganda. AIDS Care.

[36] C-h L (2006). Zhao Q, Gao E-S, Shah IH: Can the Internet be used effectively to provide sex education to young people in China?. J Adolesc Health.

[37] Zhu X, Zhang W, Operario D, Zhao Y, Shi A, Zhang Z, Gao P, Perez A, Wang J, Zaller N (2019). Effects of a mobile health intervention to promote HIV self-testing with MSM in China: a randomized controlled trial. AIDS Behavior.

[38] Odeny TA, Bailey RC, Bukusi EA, Simoni JM, Tapia KA, Yuhas K, Holmes KK, McClelland RS (2012). Text messaging to improve attendance at post-operative clinic visits after adult male circumcision for HIV prevention: a randomized controlled trial. PLoS ONE.

[39] Ybarra M, Biringi R, Prescott T, Bull SS (2012). Usability and navigability of an HIV/AIDS internet intervention for adolescents in a resource limited setting. Comput Informatics Nurs.

[40] Ybarra ML, Bull SS, Prescott TL, Korchmaros JD, Bangsberg DR, Kiwanuka JP (2013). Adolescent abstinence and unprotected sex in CyberSenga, an Internet-based HIV prevention program: randomized clinical trial of efficacy. PLoS One.

[41] Verhey IJ, Ryan GK, Scherer N, Magidson JF (2020). Implementation outcomes of cognitive behavioural therapy delivered by non-specialists for common mental disorders and substance-use disorders in low-and middle-income countries: a systematic review. Int J Mental Health Syst.

[42] Kemp CG, Jarrett BA, Kwon C-S, Song L, Jetté N, Sapag JC, Bass J, Murray L, Rao D, Baral S (2019). Implementation science and stigma reduction interventions in low-and middle-income countries: a systematic review. BMC Med.

[43] Kemp CG, Weiner BJ, Sherr KH, Kupfer LE, Cherutich PK, Wilson D, Geng EH, Wasserheit JN (2018). Implementation science for integration of HIV and non-communicable disease services in sub-Saharan Africa: a systematic review. Aids.

[44] Sabben G, Mudhune V, Ondeng'e K, Odero I, Ndivo R, Akelo V, Winskell K (2019). A Smartphone Game to Prevent HIV Among Young Africans (Tumaini): assessing intervention and study acceptability among adolescents and their parents in a randomized controlled trial. JMIR mHealth uHealth.

[45] Gardner W (2016). Native VOICES: Adapting a video-based sexual health intervention for American Indian teens and young adults using the ADAPT-ITT Model. Am Indian Alaska Native Mental Health Research.

[46] Kerrigan D, Moreno L, Rosario S, Sweat M (2001). Adapting the Thai 100% condom programme: developing a culturally appropriate model for the Dominican Republic. Culture Health Sexuality.

[47] Lauricella M, Valdez JK, Okamoto SK, Helm S, Zaremba C (2016). Culturally grounded prevention for minority youth populations: a systematic review of the literature. J Primary Prevention.

[48] Airhihenbuwa CO, Ford CL, Iwelunmor JI (2014). Why culture matters in health interventions: lessons from HIV/AIDS stigma and NCDs. Health Educ Behavior.

[49] Muula AS, Mfutso-Bengo JM (2004). Important but neglected ethical and cultural considerations in the fight against HIV/AIDS in Malawi. Nurs Ethics.

[50] Maticka-Tyndale E (2012). Condoms in sub-Saharan Africa. Sexual Health.

[51] Green LW, Glasgow RE (2006). Evaluating the relevance, generalization, and applicability of research: issues in external validation and translation methodology. Eval Health Professions.

[52] Castro FG, Barrera M, Martinez CR (2004). The cultural adaptation of prevention interventions: resolving tensions between fidelity and fit. Prevention Science.

[53] Lewis CC, Klasnja P, Powell BJ, Lyon AR, Tuzzio L, Jones S, Walsh-Bailey C, Weiner B (2018). From classification to causality: advancing understanding of mechanisms of change in implementation science. Front Public Health.

[54] Lyon AR, Koerner K (2016). User-centered design for psychosocial intervention development and implementation. Clin Psychol Sci Pract.

[55] Carroll C, Patterson M, Wood S, Booth A, Rick J, Balain S (2007). A conceptual framework for implementation fidelity. Implementation Sci.

[56] Onakomaiya D, Gyamfi J, Iwelunmor J, Opeyemi J, Oluwasanmi M, Obiezu-Umeh C, Dalton M, Nwaozuru U, Ojo T, Vieira D (2019). Implementation of clean cookstove interventions and its effects on blood pressure in low-income and middle-income countries: systematic review. BMJ Open.

[57] Keith RE, Hopp FP, Subramanian U, Wiitala W, Lowery JC (2010). Fidelity of implementation: development and testing of a measure. Implementation Science.

[58] Durlak JA, DuPre EP (2008). Implementation matters: a review of research on the influence of implementation on program outcomes and the factors affecting implementation. Am J Community Psychology.

[59] Damschroder LJ, Aron DC, Keith RE, Kirsh SR, Alexander JA, Lowery JC (2009). Fostering implementation of health services research findings into practice: a consolidated framework for advancing implementation science. Implementation Science.

[60] Wozney L, McGrath PJ, Gehring ND, Bennett K, Huguet A, Hartling L, Dyson MP, Soleimani A, Newton AS (2018). eMental healthcare technologies for anxiety and depression in childhood and adolescence: systematic review of studies reporting implementation outcomes. JMIR Mental Health.

[61] Proctor EK, Landsverk J, Aarons G, Chambers D, Glisson C, Mittman B (2009). Implementation research in mental health services: an emerging science with conceptual, methodological, and training challenges. Administration Policy Mental Health Mental Health Serv Res.

[62] Del Boca FK, McRee B, Vendetti J, Damon D (2017). The SBIRT program matrix: a conceptual framework for program implementation and evaluation. Addiction.

[63] Kemp CG, Velloza J (2018). Implementation of eHealth interventions across the HIV care cascade: a review of recent research. Current HIV/AIDS Reports.

[64] Stephani V, Opoku D, Quentin W (2016). A systematic review of randomized controlled trials of mHealth interventions against non-communicable diseases in developing countries. BMC Public Health.

[65] Louie E, Barrett EL, Baillie A, Haber P, Morley KC (2020). Implementation of evidence-based practice for alcohol and substance use disorders: protocol for systematic review. Systematic Reviews.

