# Supplementary material for: Mobile health interventions for HIV/STI prevention among youth in low- and middle-income countries (LMICs): a systematic review of studies reporting implementation outcomes
Source: Implement Sci Commun. 2021 Nov 6;2:126. doi: 10.1186/s43058-021-00230-w (PMC8572487; doi:10.1186/s43058-021-00230-w)
Supplement: Supplementary file 2 — Additional file 2. [file 43058_2021_230_MOESM2_ESM.docx]

Additional File 2: Search strategy for PubMed which was modified and used in other databases

| Category | Search Terms Combined with AND |
| --- | --- |
| Age group | (adolescent[MeSH] OR young adult[MeSH] OR adolescents OR youth OR teenager) |
| outcomes | (HIV OR AIDS OR “human immunodeficiency virus” OR AIDS OR sexual OR “sexual behavior” OR “sexual activity” OR “sexual debut” AND “transactional sex” OR STI OR STIs OR STD OR STDs OR “sexually transmitted infections” OR “sexually transmitted diseases”) |
| Mhealth | (eHealth OR e-Health OR mHealth OR m-Health OR “mobile health” OR cellphone OR smartphone OR “mobile phone” OR (cell phone[MeSH Terms] OR text messaging[MeSH Terms] OR mobile health[MeSH Terms] OR mobile phone[MeSH Terms]) |
| Intervention | ("HIV interventions" OR "HIV prevention" OR "HIV risk reduction" OR programs or interventions OR prevention OR intervention OR program OR “risk-reduction” OR “risk reduction” OR strategy) |
| Region/countries | (“developing countries” OR “developing country” OR “low-income countries” OR “developing world” OR “less developed country” OR “less developed countries” OR “poor countries” OR “middle income countries” OR “lower and middle income countries” OR “LMIC”) |
| Implementation outcomes | (implementation OR "implementation outcomes" OR "implementation research" OR reach OR effectiveness OR adoption OR uptake OR implementation OR maintenance OR acceptability OR appropriateness OR costs OR feasibility OR fidelity OR cost OR penetration OR sustainability)) |

Note: MeSH = medical subject heading
